# Factors associated with mortality when chronic betablocker therapy is withdrawn in the peri-operative period in vascular surgical patients: a matched case–control study

**Published:** 2010-04

**Authors:** Bruce M Biccard

**Affiliations:** Department of Anaesthetics, Nelson R Mandela School of Medicine and Inkosi Albert Luthuli Central Hospital, Durban, South Africa

**Keywords:** mortality, cardiac, surgery vascular, pharmacology beta-blockers

## Abstract

**Background:**

Withdrawal of chronic beta-blockade following vascular surgery is associated with peri-operative mortality. The aim of this study was to examine risk factors associated with mortality in patients where chronic beta-blockade was withdrawn.

**Methods:**

Two matched case–control studies were conducted, one of patients withdrawn from beta-blockade who survived and the other of patients who were maintained on beta-blockade and survived. Each case was matched with two controls. Three potential risk factors were analysed: the increase in heart rate postoperatively, the use of inotropes, and whether withdrawal for the first three postoperative days was more predictive than withdrawal for a single day. Multivariate conditional logistic regression was conducted.

**Results:**

The only independent predictor of in-hospital mortality was a change in the mean daily heart rate of ≥ six beats per minute from the day of surgery to the third postoperative day, or death or discharge if this happened before the third day (OR 13.7, 95% CI: 1.7–110, *p* = 0.014). The area under the curve for the receiver operating characteristic curve was 0.787.

**Conclusion:**

Use of a postoperative heart rate threshold may be clinically useful as an ‘early warning system’ in patients withdrawn from chronic beta-blockade in the peri-operative period.

## Summary

A history of chronic beta-blockade has been associated with a significantly increased incidence of peri-operative myocardial infarction in non-cardiac surgical patients [odds ratio (OR) 2.14, 95% confidence interval (CI): 1.29–3.56].^1^ Case series that have documented withdrawal of chronic beta-blockade following mixed vascular surgical procedures have reported extremely poor outcomes; with an in-hospital mortality of 24 to 50%, an early cardiovascular mortality of 29%, a peri-operative myocardial infarction rate of 50% and a one-year mortality following surgery of 38%.^2^,^3^ A meta-analysis of these two studies shows that the OR for mortality when chronic beta-blockade is withdrawn in the peri-operative period in vascular surgical patients is 26.32, 95% CI: 8.95–77.44, *p* < 0.0001.

Despite the significantly increased risk of mortality, the total number of cases reported is small (*n* = 29) and no attempt has been made to identify risk factors associated with mortality in patients in whom chronic beta-blockade is withdrawn in the peri-operative period. Chronic beta-blockade results in beta-adrenergic receptor up-regulation and a lowering of the ischaemic threshold,^1^ with myocardial ischaemia evident at lower myocardial oxygen demands. Withdrawal of chronic beta-blockade in the peri-operative period and an associated increase in heart rate therefore probably contributes to the increased all-cause and cardiovascular mortality, secondary to myocardial ischaemia. The relationship between postoperative heart rate and chronic beta-blockade withdrawal as an independent predictor of postoperative mortality following vascular surgery has recently been confirmed.^4^

The aim of this study was to examine clinically useful predictors of mortality in patients withdrawn from chronic beta-blockade in the peri-operative period. As it would be unethical to investigate this problem prospectively, a case–control study design was utilised.

## Methods

Local ethical approval was granted by the Ethics Committee of the Nelson R Mandela School of Medicine for this study. An existing database of all vascular surgical patients over 39 years of age admitted for both elective and emergency vascular surgery at Inkosi Albert Luthuli Central Hospital between June 2003 and June 2007 was used for this study. Only the most recent surgical procedure per patient is recorded in the database. Therefore the mortality reported in this article represents the mortality per patient and not per procedure. More recent data has not been included as this is prospectively being collected for a Medical Research Council-funded observational study.

Demographic data associated with peri-operative cardiac risk,^5^ and physiological^6^ and surgical procedural data^7^ associated with in-hospital mortality were extracted from the hospital computerised database. The following clinical risk factors are included in the database: history of ischaemic heart disease (or pathological Q waves on ECG), history of congestive heart failure, diabetes, serum creatinine > 180 μmol.l^-1^, age, gender, pre-operative and postoperative haemoglobin and blood glucose levels, and a history of hypertension. The vascular surgical procedural factors included the type of surgery, duration of surgical operating time, and whether surgery was undertaken out of elective surgical working hours. Therefore this study includes both elective and emergency surgical patients.

The physiological data included the mean daily heart rate (HR), systolic blood pressure (SBP) and diastolic blood pressure (DBP) calculated from recorded nurses’ observations during the hospital admission. A history of pre-operative chronic beta-blocker therapy and in-hospital drug administration was also recorded in the database. It was therefore possible to identify patients who did not have their beta-blocker therapy prescribed during their hospital admission.

The cause of in-hospital deaths reported in the database were determined using the stringent criteria of a previous study.^8^ Cardiac deaths were defined as a postoperative cardiac event being the primary event, which subsequently resulted in death. These cardiac events included cardiac arrest, myocardial infarction or cardiac failure. A primary cardiac arrest was defined as a witnessed cardiac arrest associated with ventricular fibrillation, ventricular tachycardia or asystole in a patient who was previously considered stable. Myocardial infarction was defined as postoperative clinical signs or symptoms consistent with myocardial ischaemia and either an associated diagnostic rise in troponin T or creatinine kinase-MB, or electrocardiographic (ECG) changes consistent with acute myocardial infarction. Primary postoperative cardiac failure was defined as clinical signs and symptoms consistent with acute pulmonary oedema requiring inotropic support without an obvious precipitant.

All patients in the database in whom chronic beta-blockade was not administered on any of the first three postoperative days were identified. Those patients who died during the hospital admission were identified as ‘cases’. These patients were matched in two separate case–control studies: the first matched ‘cases’ with patients who were withdrawn from chronic beta-blockade but survived, and the second matched ‘cases’ with patients who were continued on chronic beta-blockade in the peri-operative period and survived.

To identify matching patients, all the patients were stratified according to age. The matched controls for the cases that died were the two patients nearest in age to the case, who were matched for the presence or absence of the following risk factors (serum creatinine >180 μmol.l^-1^, surgery out of hours, and a mean daily postoperative SBP of > 179 or < 90 mmHg). These three risk factors were previously identified as independent predictors of all-cause in-hospital mortality in our vascular surgical patients.^4^ These three variables were chosen as two of them were the strongest predictors of mortality in the postoperative model (serum creatinine and surgery out of hours) and the third variable was known to interact with postoperative heart rate (SBP > 179 or < 90 mmHg).^4^

The reason for withdrawal of chronic beta-blockade was identified by analysis of case notes. Attention to beta-blocker-associated side effects including bradycardia, hypotension, and perioperative complications requiring inotropic support were analysed. If there was no obvious clinical indication for withdrawal, then the reason for withdrawal was classified as ‘unknown’.

## Statistical analysis

All demographic and possible risk factors were summarised by group (case versus control) using descriptive statistics. Mean [standard deviation (SD)] or median (interquartile range) was used where appropriate for continuous variables and frequency (proportion) for categorical variables. Conditional logistic regression was used to compare case and control groups for each variable and to take into account matching in the comparison. Categorical data of mortality associated with chronic beta-blockade and withdrawal of beta-blockade were analysed using the Fisher’s exact test.

Three risk factors possibly associated with mortality following withdrawal of chronic beta-blockade were examined using conditional logistic regression. Firstly, whether complete withdrawal of beta-blockers for the first three days was associated with mortality when compared with withdrawal for only a single day. Secondly, whether the administration of postoperative inotropes was associated with mortality. Thirdly, whether the change in mean daily heart rate from the day of surgery to the third postoperative day was associated with mortality. Where patients died or were discharged before the third postoperative day, mean daily heart rate for the last day in hospital was used. If the change in mean daily heart was found to an important univariate predictor, then a dichotomous heart rate variable was to be derived from constructing a receiver operating characteristic (ROC) curve and identifying the optimal cut-off point for the change in mean daily heart rate from the day of surgery to the ‘last mean daily heart rate’.

The variables with the strongest association with postoperative mortality following chronic beta-blocker withdrawal were entered into a multivariate conditional logistic regression. A *p*-value less than the Bonferroni adjusted α (0.017 = 0.05/number of possible risk factors) indicated statistical significance.

An independent samples *t*-test was conducted to compare heart rates between cases and controls as the heart rate was normally distributed. *P*-value, OR and CI are presented for all comparisons. SPSS 15.0 for Windows (6 Sept 2006) was used for data analysis.

## Results

Out of 829 vascular surgical patients, 195 patients on chronic beta-blockade were identified (23.5%). Both chronic beta-blockade and withdrawal of chronic beta-blockade were associated with significantly higher in-hospital mortality rates (Tables 1 and 2). Fifty per cent of the patients who were withdrawn from chronic beta-blockade were classified as having primary cardiac deaths.

**Table 1.** A History Of Chronic Beta-Blockade And Per Patient In-Hospital Mortality

	*Chronic beta-blockade*	*No chronic beta-blockade*	*Odds ratio (95% CI)*
Mortality	28/195 (14.4%)	54/634 (8.5%)	1.80 (1.11–2.93)

CI: confidence interval; *p* = 0.02

**Table 2.** Withdrawal Of Chronic Beta-Blockade And Per Patient In-Hospital Mortality

	*Withdrawal of chronic beta-blockade*	*Administration of chronic beta-blockade*	*Odds ratio (95% CI)*
Mortality	21/108 (19.4%)*	6/86 (7.0%)	3.22 (1.24–8.38)

*The data on one death is excluded as this patient died intra-operatively. CI: confidence interval; *p* = 0.01.

Of the 21 patients who were withdrawn from chronic betablockade and died, seven were excluded from the matched case–control studies, as four had missing haemodynamic data, and for three cases it was impossible to identify adequate controls for matching. The remaining fourteen cases were adequately matched with two sets of 28 controls. For both the case–control study of matched survivors who were withdrawn from chronic beta-blockade and the case–control study of matched survivors who continued chronic beta-blockade, 27 controls were matched on all three risk factors, and one control was matched for two risk factors. The patient characteristics of the 14 cases withdrawn from chronic beta-blockade and died are presented in Table 3. All 14 patients were withdrawn from chronic atenolol therapy.

**Table 3.** Patient Characteristics Of ‘Cases’ Withdrawn From Chronic Beta-Blockade

*Patient number*	Postoperative days withdrawn (n)*	*Reason for withdrawal of beta-blockade*	*Postoperative day of death*	*Cause of death*
1	1	Peri-operative inotropes	15	Cardiac: myocardial infarction
2	3	Unknown	26	Cardiac: cardiac arrest
3	3	Unknown	11	Non-cardiac: abdominal compartment syndrome
4	2	Unknown	2	Non-cardiac: cerebrovascular accident
5	1	Peri-operative inotropes	1	Non-cardiac: massive haemorrhage
6	3	Peri-operative inotropes	6	Cardiac: myocardial infarction
7	3	Unknown	8	Non-cardiac: respiratory failure
8	2	Unknown	2	Cardiac: myocardial infarction
9	1	Unknown	30	Cardiac: cardiac failure
10	1	Peri-operative inotropes	1	Cardiac: myocardial infarction
11	3	Unknown	3	Cardiac: myocardial infarction
12	3	Unknown	3	Non-cardiac: indeterminate
13	3	Postoperative ventilation	13	Non-cardiac: respiratory failure
14	3	Bradycardia	6	Cardiac: myocardial infarction

*Within the first three postoperative days

The demographic, clinical, surgical and physiological data of the cohorts were similar with the exception of a history of hypertension, which was more frequent in the matched control group of survivors withdrawn from chronic beta-blockade (Table 4).

**Table 4.** Demographic Data, Cardiac And Surgical Risk Factors And Postoperative Physiological Data For Case And Matched Control Cohorts. Values Are Mean (SD), Median (IQR) Or Number (Proportion)

	*Cases*	*Controls (withdrawn survivors)*	*Controls (continued survivors)*	*Matched case–control for beta-blocker-withdrawn survivors*			*Matched case control for beta-blocker-continued survivors*		
*Characteristic*	*(n = 14)*	*(n = 28)*	*(n = 28)*	p-value*	OR*	95% CI*	p-value*	OR*	95% CI*
*Pre-operative risk factors*									
Age	63.5 (± 7.5)	63.4 (± 7.0)	63.1 (± 7.7)						
Gender (male)	11 (78.6%)	15 (53.6%)	18 (64.3%)	0.11	5.72	0.66–49.4	0.33	2.30	0.43–12.24
Diabetic	3 (21.4%)	16 (57.1%)	11 (39.3%)	0.07	0.29	0.78–1.11	0.62	0.68	0.15–3.10
Hypertensive	9 (64.3%)	27 (96.4%)	24 (85.7%)	0.04	0.10	0.01–0.86	0.13	0.27	0.05–1.47
Smoker	9 (64.3%)	16 (57.1%)	13 (46.4%)	0.66	1.34	0.36–5.17	0.31	1.92	0.54–6.80
Ischaemic heart disease	10 (71.4%)	24 (85.7%)	21 (75%)	0.33	0.50	0.13–2.0	0.77	0.78	0.15–4.20
Congestive cardiac failure	0 (0%)	1 (3.6%)	1 (3.6%)	0.68	0.03	0–939855	0.68	0.26	0–939855
Stroke	2 (14.3%)	3 (10.7%)	4 (14.3%)	0.73	1.44	0.19–11.12	†		
Creatinine > 180 μmol.l^-1^	3 (21.4%)	5 (17.9%)	5 (17.9%)						
Pre-operative haemoglobin (g.dl^-1^)	8.8 (± 2.5)	12.3 (± 1.9)	13.1 (± 2.1)	0.41	0.31	0.02–5.22	0.40	0.27	0.01–5.72
Pre-operative glucose (mmol.l^-1^)	8.5 (± 3.7)	6.9 (± 2.9)	8.9 (± 4.2)	0.35	1.16	0.85–1.58	0.37	1.27	0.76–2.13
*Pre-operative chronic medical therapy*									
No statin therapy	12 (85.7%)	19 (67.9%)	16 (57.1%)	0.18	4.59	0.50–42.40	0.92	4.00	0.80–20.01
*Surgical risk factors*									
Supra-inguinal surgery	7 (50%)	17 (60.7%)	17 (60.7%)	0.51	0.63	0.17–2.42	0.51	0.63	0.17–2.42
Duration of surgery (minutes)	105 (65–190)	103 (76–149)	108 (58–164)	0.75	1.002	0.99–1.01	0.50	1.003	0.99–1.01
Surgery out of hours	1 (7.1%)	2 (7.1%)	2 (7.1%)						
*Postoperative physiological data and risk factors*									
SBP < 100 or > 179 mmHg	2 (14.3%)	4 (14.3%)	4 (14.3%)						
Postoperative haemoglobin (g.dl^-1^)	10.7 (± 3.3)	9.7 (± 2.4)	10.5 (± 2.1)	0.92	0.97	0.55–1.72	0.78	1.05	0.76–1.44
Postoperative glucose (mmol.l^-1^)	10.1 (± 3.9)	9.3 (± 4.3)	8.2 (± 3.8)	0.63	0.92	0.65–1.30	0.75	1.04	0.80–1.36

*Not reported for variables used for matching †: no model fitted; SBP: systolic blood pressure; SD: standard deviation; IQR: interquartile range.

**Fig. 1.**
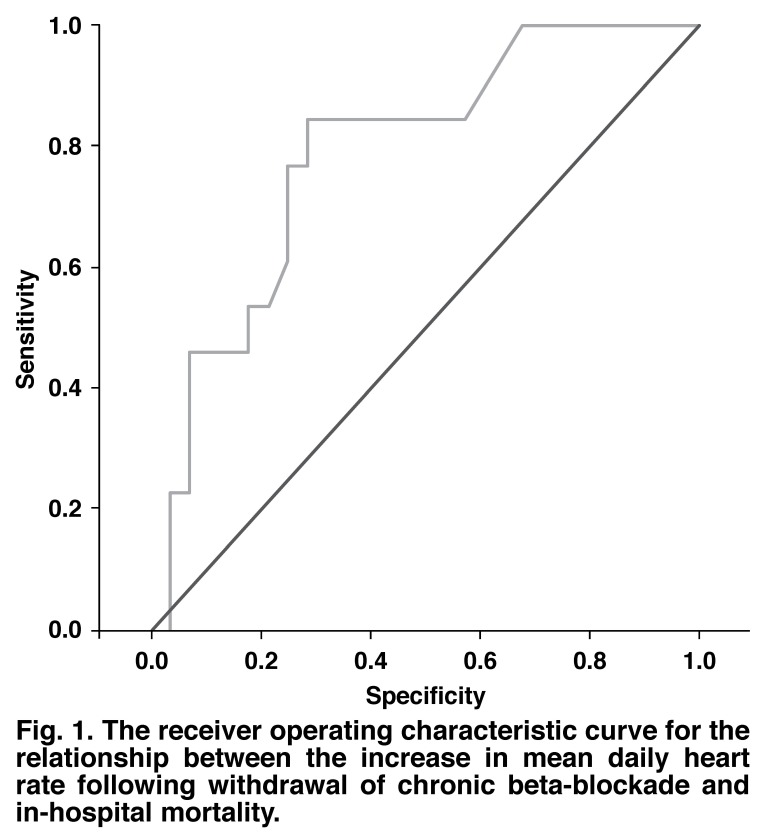
The receiver operating characteristic curve for the relationship between the increase in mean daily heart rate following withdrawal of chronic beta-blockade and in-hospital mortality.

The conditional logistic regression identified that the postoperative change in heart rate had the strongest association with postoperative mortality in the matched cohort of withdrawn survivors (Table 5). The ROC curve showed the optimal cut-off point to be a mean increase in heart rate of ≥ six beats per minute from the day of surgery to the ‘last mean daily heart rate’. This had a sensitivity of 84.6% and a specificity of 71.4% and an area under the curve of 0.787 (Fig. 1). For the cohort that was maintained on chronic beta-blockade, the same cut-off point had a sensitivity of 76.9% and a specificity of 65.4%. The area under the curve was 0.778. The optimal cut-off point for this group was a mean increase in heart rate of > 5.8 beats per minute with an 84.6% sensitivity and 65.4% specificity.

**Table 5.** Conditional Logistic Regression Of The Three Predetermined Risk Factors For Mortality Following Withdrawal From Chronic Beta-Blockade

	*Matched beta-blocker-withdrawn survivors cohort*			*Matched beta-blocker-continued survivors cohort*		
*Characteristic*	*Odds ratio*	*95% CI*	p*-value*	*Odds ratio*	*95% CI*	p*-value*
*Heart rate characteristics*						
Heart rate difference: day of surgery to last HR	1.10	1.01–1.18	0.03	1.09	1.004–1.18	0.04
Mean HR increase of ≥ six beats per minute from day of surgery*	13.66	1.70–110.09	0.014	6.06	1.27–28.93	0.024
*Beta-blocker administration*						
No beta-blocker administration in first three days versus any administation	4.92	1.01–23.97	0.05	169.5	0.9–32943	0.06
*Inotrope administration*						
Institution of peri-operative inotropic support	4.00	0.73–21.84	0.11	8.00	0.89–71.6	0.06

CI: confidence interval; HR: heart rate. *Optimal cut-off point from receiver operating characteristic curve from the withdrawn survival cohort.

No interaction was demonstrated between an increase in heart rate of ≥ six beats per minute and non-administration of beta-blockade within the first three postoperative days. Entering both these variables into the multivariate analysis found only the increase in heart rate to be independently associated with postoperative mortality following withdrawal of chronic beta-blockade in vascular surgical patients (OR 13.7, 95% CI: 1.7–110, *p* = 0.014). In the case–control study of patients maintained on chronic beta-blockade, neither the withdrawal of chronic betablockade for all three postoperative days nor the increase in heart rate were independent predictors of mortality. The heart rate characteristics of the cases and controls of the case–control study of patients withdrawn from chronic beta-blockade are presented in Table 6.

**Table 6.** Heart Rate Characteristics Of Matched Case–Control Study Of Patients Withdrawn From Chronic Beta-Blockade

	*Mean HR (SD)*		t*-test*	
	*Day of surgery*	*Last HR*	*Day of surgery*	*Last HR*
Died	72 (18)	92 (21)	0.66	0.047
Survived	75 (14)	80 (18)		

HR: heart rate; SD: standard deviation; last HR: mean heart rate on third postoperative day or last day in hospital if earlier than the third postoperative day.

## Discussion

The sample size of vascular surgical patients who were withdrawn from chronic beta-blockade in this study was nearly 1.5 times greater than the total number of cases previously reported in the published literature. This study therefore adds substantially to the data on this important peri-operative problem. It confirms that withdrawal of chronic beta-blockade is potentially lethal.

It is, however, the first study to demonstrate that withdrawal of a single dose of beta-blocker within the first 72 hours postoperatively is significantly associated with in-hospital mortality. It is also the first study to specifically address predictors of mortality in patients who are withdrawn peri-operatively from chronic beta-blockade, and to confirm that the response of the heart rate between the day of surgery and the third postoperative day is independently associated with mortality.

This study therefore suggests that rate-mediated ischaemia is critical to survival once beta-blockers are withdrawn in the peri-operative period.^1^ An increase in the mean heart rate of ≥ six beats per minute following withdrawal of chronic beta-blockade was found to be independently associated with peri-operative mortality in vascular surgical patients. The positive likelihood ratio (LR) is 3 and the negative LR is 0.2. For a test to be clinically useful, it should have a positive LR exceeding 5 to 10 and a negative LR of less than 0.2.^9^,^10^ Based on the heart rate threshold identified in this study and the associated LR, we can conclude that patients who have a heart rate increase of less than six beats per minute following withdrawal of chronic beta-blockade are likely to survive, as the LR is within the clinically useful range. When the heart rate increases by at least six beats per minute, then the discrimination is not as accurate.

Therefore although this heart rate increase is an independent predictor of mortality when chronic beta-blockade is withdrawn, it is still clinically inaccurate, reflecting the uncertainty associated with this predictor. This poor positive discrimination is not an unusual problem with peri-operative risk prediction.^9^ Indeed, none of the positive results of the current special investigations for vascular surgery appear to be accurate enough for positive discrimination.^11^

The poor positive discrimination of this heart rate threshold should however not be considered an important limitation, especially when one considers how this heart rate threshold may impact on peri-operative anaesthetic practice. When patients on chronic beta-blockade present for surgery, keeping the heart rate below the ischaemic threshold is a central tenant. This target could be identified by determining the ischaemic threshold from pre-operative Holter monitoring or by arbitrarily selecting a heart rate threshold.^1^ Certainly, with a negative LR of 0.2, the heart rate threshold presented in this article is clinically useful. If an anaesthetist could successfully control the heart rate below this threshold, then it would probably be unnecessary to conduct a pre-operative Holter in order to determine an ischaemic threshold.

However, what should we do with patients in whom chronic beta-blockade is withdrawn and who have an increase in heart rate ≥ six beats per minute? As we cannot accurately predict which patients will die using this threshold, but as we do know that this group is at a significantly increased risk of in-hospital mortality, we could use this threshold as an ‘early warning system’. It would be reasonable to attempt to re-institute betablockade as soon as possible if not contra-indicated. Transfer of the patient to a high-care facility, serial monitoring of troponins, repeat ECG^12^ and an attempt to institute other negatively chronotropic therapy if beta-blockade is contra-indicated should all be considered.

Although withdrawal of chronic beta-blockade is clearly undesirable, 1-3 it does occur, either inadvertently, or as a considered decision based on perceived contra-indications or peri-operative complications considered to be related to beta-blocker administration. The findings of this study may be useful in identifying patients at risk and initiating management and therapy that may decrease the high associated morbidity and mortality.

There were limitations to this study. One-third of the patients who were withdrawn from beta-blockade were excluded from the study because of missing data. It is possible that these patients could have had a meaningful impact on the statistical analysis. In over 50% of the patients who had beta-blockers withdrawn and died, there was no obvious reason for the withdrawal (Table 3). Clearly, such a high withdrawal rate with no obvious indication needs to be studied and the reasons for withdrawal elicited, as omission of chronic beta-blockade is potentially life threatening.

It is important to note that even in chronically beta-blocked patients, there is a significant circadian rhythm to the mean heart rate with the nocturnal heart rate being lower than the daily heart rate.^13^ The ischaemic threshold is significantly decreased in the early morning.^14^ It is likely that a number of factors contribute to increased myocardial oxygen demand in the morning, including heart rate, blood pressure, autonomic and humeral physiological changes.^15^ The use of a mean daily heart rate, as used in this study, has limitations due to the variation of the ischaemic threshold through the course of a day. It is likely that the optimal cut-off point for the change in heart rate is lower than six beats per minute in the morning and possibly higher in the evening. This will, however, only be adequately addressed with a perioperative study using continuous Holter monitoring.

We also did not have data on the dosage of chronic beta-blockade that all the patients in this study received, which may be an important determinant of the ischaemic threshold.^16^ Higher doses are associated with a lower ischaemic threshold, making it possible that an increase in heart rate of ≥ six beats per minute may be too high for patients on a high dose of chronic beta-blockade. This then is another area that requires further investigation, particularly if a heart rate-based cut-off point is to be used.

Furthermore, there were statistical limitations to this study. The large confidence intervals for the OR of mortality associated with an increase in postoperative heart rate (95% CI: 1.7–110) are of limited clinical utility. There are two possible explanations for the large confidence interval: firstly, the sample size was small and secondly, it is possible that the retrospective data collection in this study may have increased the variability of the heart rate data collected. A small sample size results in less precision around an estimate of risk (and hence a larger confidence interval) and increased variability within a group decreases the reliability of the findings, and again, increases the width of the confidence intervals.^17^ It is important to note that despite the wide confidence intervals in this study, the postoperative increase in heart rate was still significantly associated with mortality. It appears therefore that this was an important observation, although it is difficult to precisely quantify its risk.

Is it clinically plausible that a heart rate change of only six beats per minute can explain an associated increased mortality? It probably is, for a number of reasons. Firstly, a Holter study of patients with stable coronary artery disease patients receiving beta-blocker therapy for two weeks has shown that silent myocardial ischaemia is evident with an increase in heart rate of as little as 12.3 (± 1.4) beats per minute from the mean resting heart rate, immediately prior to the onset of myocardial ischaemia.^18^ Secondly, beta-blockade has been shown to significantly decrease the ischaemic threshold,^16^ and that increasing doses of beta-blocker are associated with further significant reduction in the ischaemic threshold.^16^ Thirdly, the ischaemic threshold is associated with the duration of the heart rate increase, where a lower ischaemic threshold is evident if the heart rate increase is prolonged.^19^ Furthermore, there is an increased frequency of myocardial ischaemia at lower heart rates in patients on beta-blocker therapy.^19^

As heart rate tends to increase in the first three postoperative days,^20^ it is likely therefore that a heart rate change of as little as six beats per minute may be associated with significant myocardial ischaemia in vascular surgical patients who were chronically beta-blocked and then withdrawn from therapy.

## Conclusion

An increase in the mean daily heart rate of ≥ six beats per minute is independently associated with in-hospital mortality following the withdrawal of chronic beta-blockade in vascular surgical patients. It is possible that re-institution of beta-blockade or other techniques of rate control in these patients may improve survival. The relationship between the postoperative heart rate and both the dose of chronic beta-blockade and circadian rhythm needs further investigation.

## References

[1] Giles JW, Sear JW, Foex P (2004). Effect of chronic beta-blockade on perioperative outcome in patients undergoing non-cardiac surgery: an analysis of observational and case control studies.. Anaesthesia.

[2] Shammash JB, Trost JC, Gold JM, Berlin JA, Golden MA, Kimmel SE (2001). Perioperative beta-blocker withdrawal and mortality in vascular surgical patients.. Am Heart J.

[3] Hoeks SE, Scholte Op Reimer WJ, van Urk H, Jorning PJ, Boersma E, Simoons ML (2007). Increase of 1-year mortality after perioperative betablocker withdrawal in endovascular and vascular surgery patients.. Eur J Vasc Endovasc Surg.

[4] Biccard BM, Pooran RR (2008). Validation of a model to predict all-cause in-hospital mortality in vascular surgical patients.. Cardiovasc J Afr.

[5] Lee TH, Marcantonio ER, Mangione CM, Thomas EJ, Polanczyk CA, Cook EF (1999). Derivation and prospective validation of a simple index for prediction of cardiac risk of major noncardiac surgery.. Circulation.

[6] Goldhill DR, McNarry AF (2004). Physiological abnormalities in early warning scores are related to mortality in adult inpatients.. Br J Anaesth.

[7] Leung JM, Dzankic S (2001). Relative importance of preoperative health status versus intraoperative factors in predicting postoperative adverse outcomes in geriatric surgical patients.. J Am Geriatr Soc.

[8] Biccard BM, Bandu R (2007). Clinical risk predictors associated with cardiac mortality following vascular surgery in South African patients.. Cardiovasc J Afr.

[9] Ridley S (2003). Cardiac scoring systems – what is their value?. Anaesthesia.

[10] Coetzee JF (2004 (November)). Evaluating diagnostic tests.. S Afr J Anaesthes Analges.

[11] Kertai MD, Boersma E, Bax JJ, Heijenbrok-Kal MH, Hunink MG, L’Talien G J (2003). A meta-analysis comparing the prognostic accuracy of six diagnostic tests for predicting perioperative cardiac risk in patients undergoing major vascular surgery.. Heart.

[12] Rinfret S, Goldman L, Polanczyk CA, Cook EF, Lee TH (2004). Value of immediate postoperative electrocardiogram to update risk stratification after major noncardiac surgery.. Am J Cardiol.

[13] Burger AJ, Kamalesh M (1999). Effect of beta-adrenergic blocker therapy on the circadian rhythm of heart rate variability in patients with chronic stable angina pectoris.. Am J Cardiol.

[14] Figueras J, Lidon RM (1995;). Early morning reduction in ischemic threshold in patients with unstable angina and significant coronary disease.. Circulation.

[15] Li JJ (2003). Circadian variation in myocardial ischemia: the possible mechanisms involving in this phenomenon.. Med Hypoth.

[16] Tzivoni D, Medina A, David D, Barzilai Y, Brunel P (1998). Effect of metoprolol in reducing myocardial ischemic threshold during exercise and during daily activity.. Am J Cardiol.

[17] Gardner MJ, Altman DG (1989). Statistics with Confidence – Confidence Intervals and Statistical Guidelines..

[18] Stone PH, Gibson RS, Glasser SP, DeWood MA, Parker JD, Kawanishi DT (1990). Comparison of propranolol, diltiazem, and nifedipine in the treatment of ambulatory ischemia in patients with stable angina. Differential effects on ambulatory ischemia, exercise performance, and anginal symptoms. The ASIS Study Group.. Circulation.

[19] McLenachan JM, Weidinger FF, Barry J, Yeung A, Nabel EG, Rocco MB (1991). Relations between heart rate, ischemia, and drug therapy during daily life in patients with coronary artery disease.. Circulation.

[20] Poldermans D, Boersma E, Bax JJ, Thomson IR, van de Ven LL, Blankensteijn JD (1999). The effect of bisoprolol on perioperative mortality and myocardial infarction in high-risk patients undergoing vascular surgery. Dutch Echocardiographic Cardiac Risk Evaluation Applying Stress Echocardiography Study Group.. N Engl J Med.

